# Puerarin Induces Mitochondria-Dependent Apoptosis in Hypoxic Human Pulmonary Arterial Smooth Muscle Cells

**DOI:** 10.1371/journal.pone.0034181

**Published:** 2012-03-23

**Authors:** Chan Chen, Chun Chen, Zhiyi Wang, Liangxing Wang, Lehe Yang, Minjiao Ding, Cheng Ding, Yu Sun, Quan Lin, Xiaoying Huang, Xiaohong Du, Xiaowei Zhao, Chuangyi Wang

**Affiliations:** 1 Department of Geriatric Medicine, The First Affiliated Hospital of Wenzhou Medical College, Wenzhou, Zhejiang, China; 2 Department of Respiratory Medicine, The First Affiliated Hospital of Wenzhou Medical College, Wenzhou, Zhejiang, China; 3 Department of Emergency Medicine, The Second Affiliated Hospital of Wenzhou Medical College, Wenzhou, Zhejiang, China; University of Giessen Lung Center, Germany

## Abstract

**Background:**

Pulmonary vascular medial hypertrophy in hypoxic pulmonary arterial hypertension (PAH) is caused in part by decreased apoptosis in pulmonary artery smooth muscle cells (PASMCs). Puerarin, an isoflavone purified from the Chinese medicinal herb kudzu, ameliorates chronic hypoxic PAH in animal models. Here we investigated the effects of puerarin on apoptosis of hypoxic human PASMCs (HPASMCs), and to determine the possible underlying mechanisms.

**Methodology/Principal Findings:**

HPASMCs were cultured for 24 h in normoxia or hypoxia (5% O_2_) conditions with and without puerarin. Cell number and viability were determined with a hemacytometer or a cell counting kit. Apoptosis was detected with a TUNEL test, rhodamine-123 (R-123) fluorescence, a colorimetric assay, western blots, immunohistochemical staining and RT-PCR. Hypoxia inhibited mitochondria-dependent apoptosis and promoted HPASMC growth. In contrast, after puerarin (50 µM or more) intervention, cell growth was inhibited and apoptosis was observed. Puerarin-induced apoptosis in hypoxic HPASMCs was accompanied by reduced mitochondrial membrane potential, cytochrome c release from the mitochondria, caspase-9 activation, and Bcl-2 down-regulation with concurrent Bax up-regulation.

**Conclusions/Significance:**

Puerarin promoted apoptosis in hypoxic HPASMCs by acting on the mitochondria-dependent pathway. These results suggest a new mechanism of puerarin relevant to the management of clinical hypoxic pulmonary hypertension.

## Introduction

Pulmonary vascular remodeling, the pathological basis of pulmonary arterial hypertension (PAH), is characterized by changes in pulmonary vascular structure associated with medial hypertrophy, which is due partly to the inhibition of pulmonary artery smooth muscle cell (PASMC) apoptosis [1]–[4]. Induction of PASMC apoptosis results in progressive regression of pulmonary vascular medial hypertrophy [5]–[9]. The role of mitochondria in pulmonary vascular biology, and PAH specifically, has generated substantial interest in recent years. Hypoxia, a common pathophysiologic state, is one of the most important pathological factors that induces pulmonary hypertension (PH), and has a mitogenic effect on PASMCs *in vitro* [10], [11].

Mitochondria-dependent apoptosis has been identified as a potential target for PAH [7]. The mitochondrial apoptotic pathway is mediated by Bcl-2 family proteins, which have a central regulatory role in deciding the fate of cells through interactions between pro- and anti-apoptotic members [12]. The breakdown of the mitochondrial membrane potential (Δψ_m_) is an early stage of the apoptotic process, and precedes nuclear disintegration [13]. Δψ_m_ disruption is mediated by the opening of permeability transition (PT) pores in the inner mitochondrial membrane, leading to matrix swelling, outer membrane rupture, and the release of apoptotic signaling molecules, such as cytochrome c, from the intermembrane space [14], [15], [16]. An increase in cytosolic cytochrome c, in association with Apaf-1, is a trigger for the activation of caspase-9, which then activates cysteine proteases (e.g., caspases-3, -5, and -7) that are central executioners of the apoptotic pathway [17]. Mitochondria-dependent apoptosis was suppressed in hypoxia-induced human PASMCs (HPASMCs) *in vitro* [18] and in PASMCs from patients and rats with PAH [8].

Puerarin [7-hydroxy-3-(4-hydroxyphenyl)-1-benzopyran-4-one 8-(*β*-D-glucopyranoside); C_21_H_20_C_9_], the major active ingredient extracted from the Chinese medicinal herb kudzu, has multiple pharmacological activities [19]. It has long been used to treat angiocardiopathy, including coronary artery diseases (CAD) [20] and hypertension [21]. Additionally, puerarin induces apoptosis in HT-29 colon cancer cells [22] and breast cancer cells [23]. Recent findings [24] suggest that puerarin can improve pulmonary vascular remodeling in rats with PH, but the underlying mechanisms, especially the effect on the growth of HPASMCs, remain unclear.

In this study, we investigated HPASMC apoptosis in hypoxic conditions, and puerarin-induced apoptosis in hypoxic HPASMCs.

## Materials and Methods

### Materials

HPASMCs and a Smooth Muscle Cell Growth Medium-2 (SmGM-2) kit were purchased from PromoCell GmbH (Heidelberg, Germany). Injectable puerarin was purchased from Zhengzhou Lingrui Pharmaceutical Co., Ltd. (Henan, China). Trypan blue and rhodamine-123 (R-123) were purchased from Sigma-Aldrich (St. Louis, MO, USA). An In-Situ Cell Death Detection Kit was purchased from Roche Diagnostics (Penzberg, Germany). A Cell Counting Kit-8 (CCK-8) was from Dojindo Laboratories (Kumamoto, Japan). A Cytochrome c Releasing Apoptosis Assay Kit and Caspase-9 Colorimetric Assay Kit were purchased from Biovision (Mountain View, CA, USA). A mouse anti-α-actin monoclonal antibody, rabbit anti-Bcl-2 and anti-Bax polyclonal antibody were purchased from Santa Cruz Biotechnology (Santa Cruz, CA, USA). A mouse anti-cytochrome oxidase IV (COXIV) monoclonal antibody was purchased from Abcam (Cambridge, UK). Horseradish peroxidase (HRP) -conjugated monoclonal mouse anti-glyceraldehyde 3-phosphate dehydrogenase (GAPDH) was purchased from Kangchen Bio-tech (Shanghai, China). Peroxidase-labeled affinity purified antibody to mouse IgG (H+L) was purchased from KPL (Gaithersburg, MD, USA). Alexa fluor 488-labeled goat anti-mouse IgG (H+L) was purchased from Molecular Probes (Eugene, OR, USA). An ImmPRESS reagent anti-rabbit Ig kit was purchased from Vector Laboratories (Burlingame, CA, USA). Dulbecco's modified Eagle's medium (DMEM; high glucose), Trizol and SYBR Green were purchased from Invitrogen (Carlsbad, CA, USA). BeyoECL Plus was purchased from Beyotime Bio-tech (Jiangsu, China).

### Cell culture and treatments

Recovery of cryopreserved HPASMCs was performed according to supplier instructions. HPASMCs were cultured in SmGM-2 supplemented with 5% fetal bovine serum (FBS), 2 ng/ml basic fibroblast growth factor, 5 µg/ml insulin, and 0.5 ng/ml epidermal growth factor (complete medium) maintained at 37°C with 5% CO_2_. The medium was changed every two days. After reaching 80 ­ 90% confluency, cells were washed with phosphate-buffered saline (PBS; pH 7.4) and treated with 0.05% trypsin-EDTA for further passage.

Cells were used at passages 4 ­ 10 for experiments. No changes in cell morphology were noted and cells were positive for α-smooth muscle actin by immunofluorescence. For all experiments, cells were made quiescent by incubation in serum-free DMEM for 24 h. Hypoxia treatment was performed in a CO_2_-N_2_ incubator (Heraeus, Germany) at 5% O_2_/5% CO_2_/90% N_2_. HPASMCs were divided into 7 groups: (1) Normal (N), in which cells were cultured in serum-free DMEM under normoxia (21% O_2_, 5% CO_2_) for 24 h; (2) Normoxia + puerarin (NP), in which cells were cultured in serum-free DMEM with puerarin (50 µM) for 24 h under normoxia conditions; (3) Hypoxia (H), in which cells were cultured in serum-free DMEM under hypoxia (5% O_2_, 5% CO_2_) for 24 h; (4) Hypoxia + 1 µM puerarin (HP1), in which cells were cultured in serum-free DMEM with puerarin (1 µM) for 24 h under hypoxia; (5) Hypoxia +10 µM puerarin (HP10); (6) Hypoxia +50 µM puerarin (HP50); and (7) Hypoxia +100 µM puerarin (HP100).

### Determination of cell proliferation

Cell proliferation was quantified by counting the cell number directly and with CCK-8. Briefly, HPASMCs were seeded in 6-well microplates at 5×10^4^ cells/ml. Cell number was determined with a hemacytometer using 0.45% trypan blue. For CCK-8 measurements, cells were plated in flat-bottomed 96-well microplates at 1×10^4^ cells/well and incubated in complete medium at 37°C in 5% CO_2_/95% humidity air for 24 h (3 wells in each group), then treated as described above. CCK-8 (10 µl/well) was added to the wells at the end of the experiment. After incubation at 37°C for 2 h, the absorbance of each well was determined using a microplate reader (ELX800, BioTek Instruments, Winooski, VT, USA) at 450 nm.

### Detection of apoptosis

After treatment, the cells were fixed with 4% paraformaldehyde in PBS for 1 h at 25°C. Cells were subjected to a terminal deoxynucleotidyl transferase-mediated deoxyuridine triphosphate nick end labeling (TUNEL) assay using the In-Situ Cell Death Detection Kit according to the manufacturer's instructions. TUNEL-positive cells were colored using diaminobenzidine (DAB) as the chromogen, and counterstained with hematoxylin. The percentage of TUNEL-positive cells was assessed in five randomly selected fields each section.

### Measurement of mitochondrial membrane potential (Δψm)

Cells grown on coverslips (20 mm) were incubated with R-123 (5 µg/ml) for 30 min at 37°C. R-123 was taken up selectively by mitochondria in a manner dependent on Δψ_m_. R-123 fluorescence was excited at 488 nm and measured at 530 nm with a laser scanning confocal microscope (Olympus, Tokyo, Japan). In isolated mitochondria, the relationship between R-123 fluorescence and Δψ_m_ is linear. Disruption of Δψ_m_ was associated with a decrease in fluorescence due to a lack of R-123 retention. The fluorescence intensity of R-123 was analyzed with Image-Pro plus (v. 6.0) software (Media Cybernetics, Bethesda, MD, USA). Each group consisted of three cover slips randomly selected for analysis.

### Western blotting analysis

Mitochondrial protein and cytosolic protein were isolated using the Cytochrome c Releasing Apoptosis Assay kit according to the manufacturer's instructions. The proteins were quantified by the Bradford method with bovine serum albumin (BSA) as the standard. Equal amounts of proteins (20 µg) were separated by 12% SDS-PAGE and transferred onto a polyvinylidene fluoride (PVDF) membrane (Millipore, Billerica, MA, USA). Blots were blocked in Tris-buffered saline with Tween 20 (TBST) containing 5% BSA for 2 h. Subsequently, the membrane was incubated overnight at 4°C with specific primary antibodies: mouse anti-cytochrome c monoclonal antibody (1 µg/ml) and mouse anti-COXIV monoclonal antibody (0.5 µg/ml). The membranes were washed with TBST buffer, and then incubated with HRP-labeled anti-mouse Ig (0.1 µg/ml). Detection of immunoreactive bands were performed using BeyoECL Plus reagents. After scanning the X-ray film, the optical density of the immunoblots was calculated with the Quantity one −4.6.2 software (Bio-Rad Laboratories, Hercules, CA, USA). A membrane incubated with a HRP-conjugated monoclonal anti-GAPDH (0.1 ug/ml) served as the control.

### Caspase-9 activity assay

Caspase-9 activity was measured through cleavage of the colorless caspase-9 specific substrate acetyl-Leu-Glu-His-Asp-p-nitroanilide (Ac-LEHD-pNA) to release the chromophore p-nitroaniline (pNA). The assay was performed using the Caspase-9 Colorimetric Assay Kit according to the instructions. Briefly, cells were lysed with chilled lysis buffer followed by centrifugation for 1 min at 10,000×*g*, and the supernatants (cytosolic extracts) were incubated with LEHD-pNA at 37°C for 2 h. The absorbance of pNA (405 nm) was measured for each sample on a microplate reader (ELX800, BioTek Instruments, Winooski, VT, USA).

### Immunohistochemical detection of Bcl-2 and Bax proteins expression

Immunohistochemistry was used to detect the expression of Bcl-2 and Bax proteins using streptavidin-peroxidase (SP). After treatment, cells were fixed with 4% paraformaldehyde in PBS at room temperature for 20 min. Endogenous peroxidase in the section was blocked by 3% hydrogen peroxide for 15 min at room temperature. The sections were blocked in PBS containing 10% normal horse serum for 30 min at room temperature. The cells were incubated with the primary antibody, a rabbit polyclonal antibody against Bcl-2 or Bax protein (1∶100 dilution), at 4°C overnight. After washing three times with PBS, sections were incubated with ImmPRESS reagent anti-rabbit Ig for 30 min. They were then washed three times with PBS. Finally, specimens were incubated in DAB for 5 min, followed by hematoxylin counterstaining. Images from the entire sections were acquired using a digital camera (Nikon, Tokyo, Japan), and were analyzed with Image-Plus (v. 6.0). The average optical density, representing protein expression, was counted in four random fields (obtained using a 20× objective) each cover slip.

### Detection of Bcl-2 and Bax mRNA by real-time quantitative reverse transcription -polymerase chain reaction (RT-PCR)

Total RNA was isolated from the cells using Trizol reagent according to the manufacturer's protocol. RNA concentration and purity were measured with a spectrophotometer at A260 and A260/280, respectively. The reverse transcription reaction was performed with the Gene Amp PCR System 9700 (Applied Biosystems, Carlsbad, CA, USA) for the first-strand cDNA synthesis. RT-PCR was performed on a Rotor-Gene 3000 apparatus (Corbett Research Company, Sydney, Australia) with SYBR Green fluorescent dye. The sequences of primers used are as follows: Bax, forward: 5′-CCTTTTCTACTTTGCCAGCAAAC-3′ and reverse: 5′- GAGGCCGTCCCAACCAC-3′; Bcl-2, forward: 5′- ATGTGTGTGGAGAGCGTCAACC-3′ and reverse: 5′- TGAGCAGAGTCTTCAGAGACAGCC-3′; GAPDH, forward: 5′- CTCTCTGCTCCTCCTGTTCGAC-3′ and reverse: 5′- TGAGCGATGTGGCTCGGCT -3′. The PCR conditions were as follows: a pre-denaturing at 95°C for 5 min, followed by 35 cycles of denaturation at 95°C for 10 s, annealing/extension at 59°C for 15 s, and then 72°C for 20 s. A relative amount for each gene examined was obtained from a standard curve generated by plotting the cycle threshold value against the concentration of a serially diluted RNA sample expressing the gene of interest. This amount was normalized to the level of GAPDH mRNA.

### Statistical analyses

Data are expressed as the mean ± the standard deviation (SD) with the number (*n*) of experiments. Differences between data sets were assessed by one-way ANOVA followed by a Student-Newman-Keuls test. A *P*<0.05 was considered statistically significant.

## Results

### The effect of hypoxia and puerarin on the cell growth of HPASMCs

**Figure 1:**
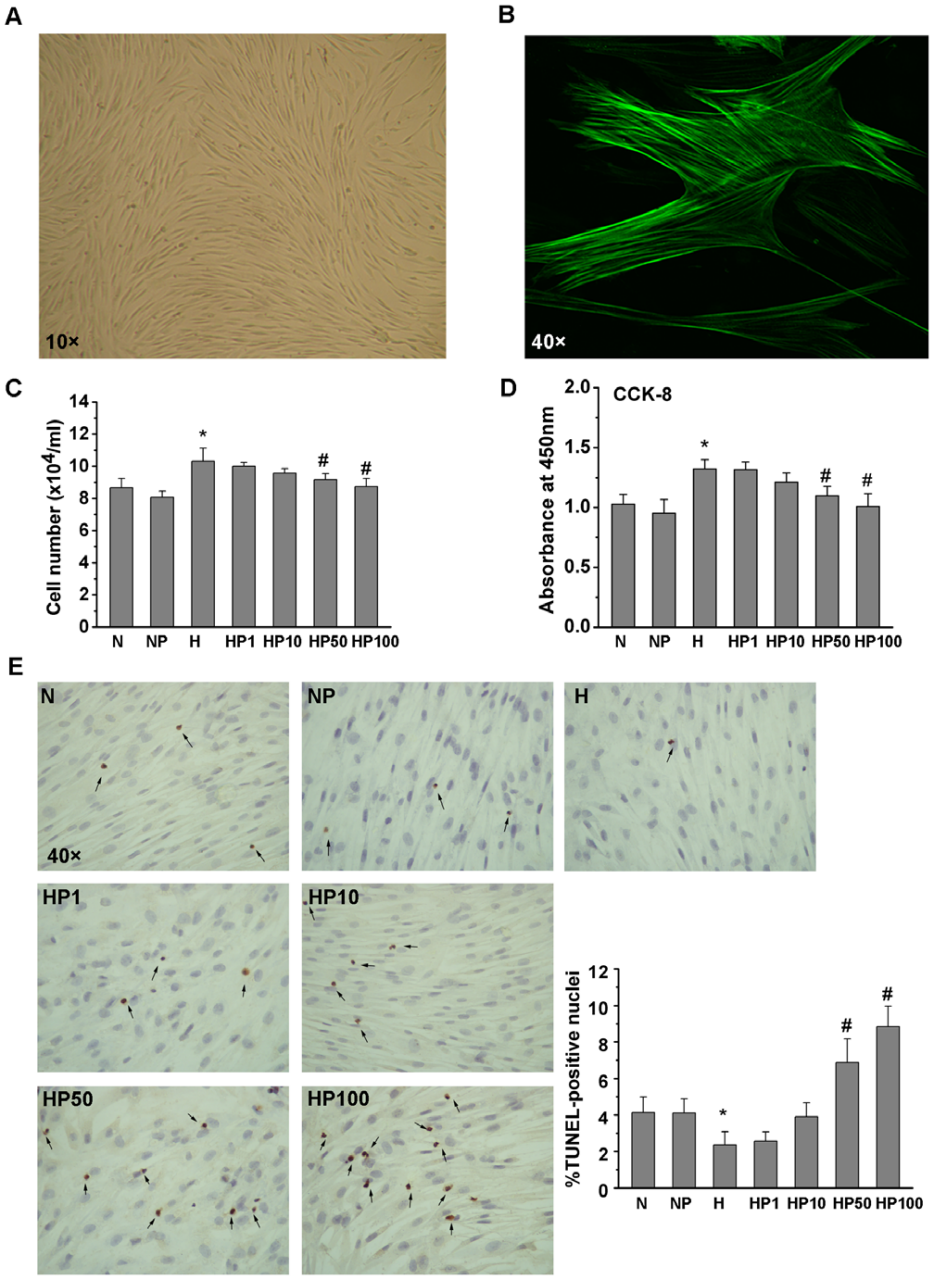
HPASMCs morphology and cell growth. A) Typical “hill and valley” appearance of HPASMCs under phase contrast microscope. B) Immunofluorescent identification of α- smooth muscle actin was positive. C) Effect of hypoxia and puerarin on cell number. HPASMCs were cultured for 24 h in normoxia or hypoxia (5% O_2_) with and without puerarin. Cell number was determined with a hemacytometer using 0.45% trypan blue. D) Cell viability was tested with CCK-8. E) Apoptosis was detected with a TUNEL assay. * *P*<0.05 vs. the N group; # *P*<0.05 vs. the H group; n = 3.

As shown in Figure 1A and B, confluent HPASMCs manifested typical “hill and valley” features, and were positive for α-smooth muscle actin by immunofluorescent staining. Compared to the N group, cell number was increased in the H group (10.33±0.80 vs. 8.67±0.58 cells/ml, respectively; Figure 1C). Puerarin significantly inhibited HPASMC growth stimulated by hypoxia in a dose-dependent manner. CCK-8 confirmed the effect of hypoxia (H group; Figure 1D). A significant decrease in cell viability was observed upon treatment with puerarin in a dose-dependent manner (50 to 100 µM), however, 50 µM puerarin had no significant effect on cell number or viability in normoxic HPASMCs.

### Hypoxia and puerarin affect HPASMC apoptosis

To determine whether puerarin inhibited cell viability by inducing apoptosis in hypoxic HPASMCs, a TUNEL assay was used. As shown in Figure 1E, the percentage of TUNEL-positive cells decreased in the H group, compared to the N group (2.35±0.73% vs. 4.13±0.85%, respectively; *P*<0.05). In addition, there were significant increases in TUNEL-positive cells at 50 and 100 µM puerarin after 24 h exposure. Indeed, a 2.93 and 3.76-fold, respectively, increase in TUNEL-positive cells over the H group was observed. However, 50 µM puerarin had no significant effect on apoptosis in normoxic HPASMCs.

### Hypoxia and puerarin alter the Δψ_m_ of HPASMCs

**Figure 2:**
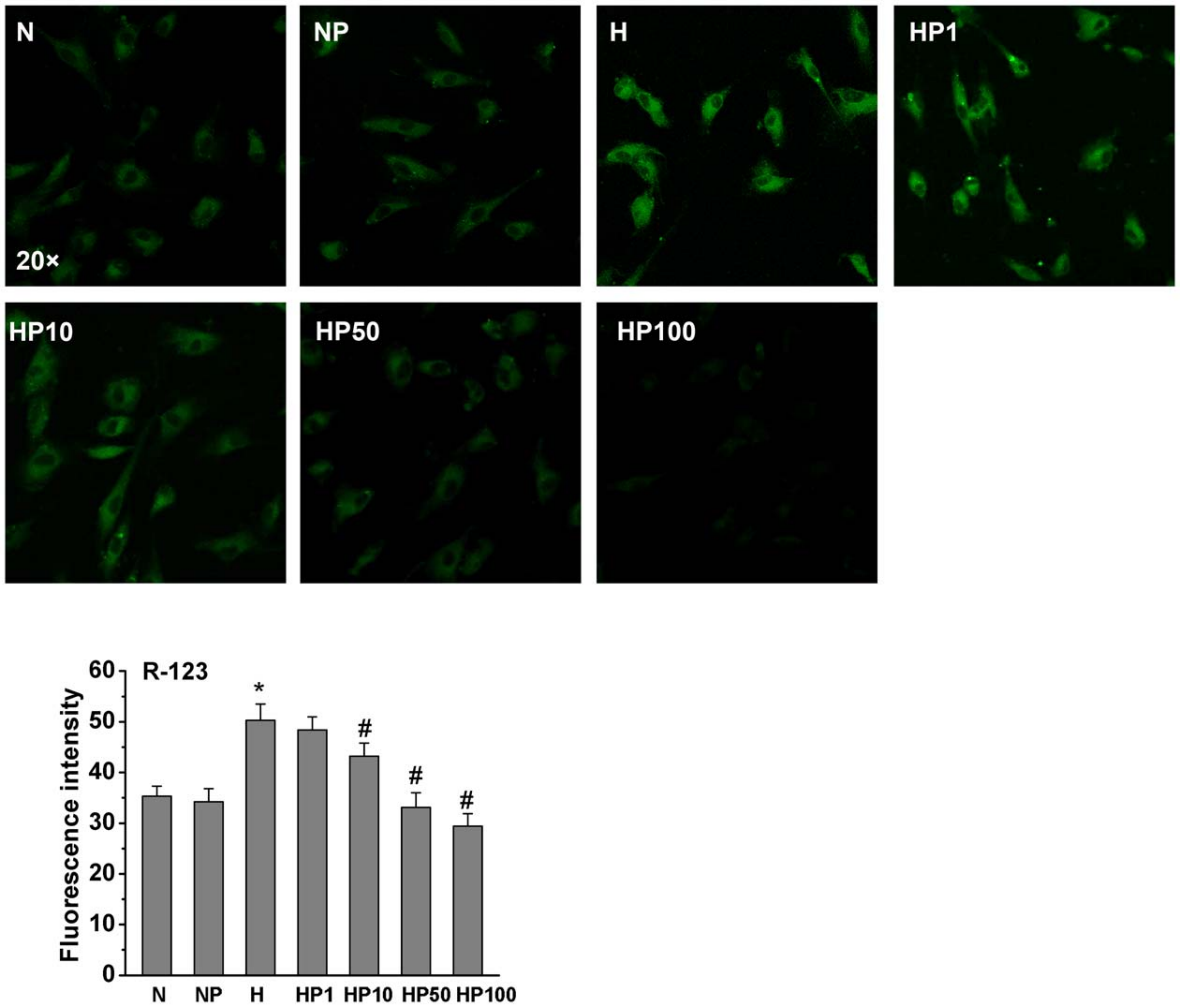
Hypoxia increased Δψ_m_ in HPASMCs, an effect reversed by puerarin. Δψ_m_ was measured with R-123. The H group had increased Δψ_m_ compared to the N group, while puerarin restored Δψ_m_ in hypoxic HPASMCs. * *P*<0.05 vs. the N group; # *P*<0.05 vs. the H group; n = 3.

As shown in Figure 2, the H group had increased Δψ_m_ compared to the N group. The treatment of hypoxic HPASMCs with puerarin markedly decreased the Δψ_m_ in a dose-dependent manner. Puerarin (50 µM) did not affect Δψ_m_ in normoxic HPASMCs.

### Puerarin induced cytochrome c release and caspase-9 activation

**Figure 3:**
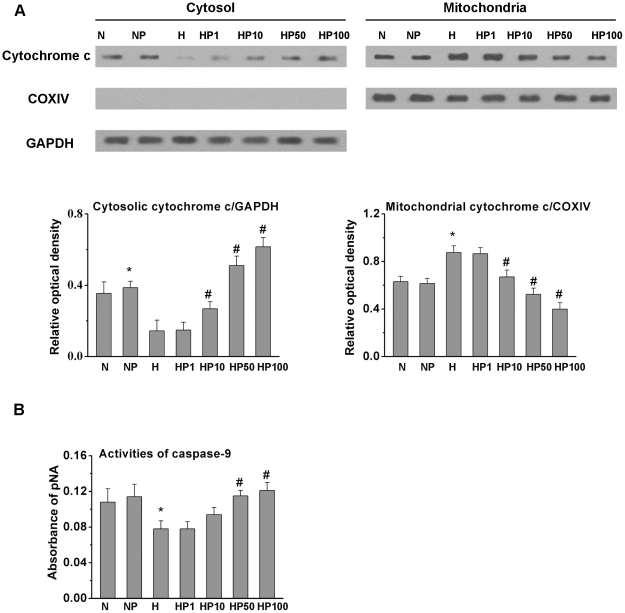
Puerarin induced cytochrome c release and caspase-9 activation in hypoxic but not normoxic HPASMCs. A) Cytochrome c distribution. COXIV and GAPDH detection were used to determine the purity of the cytoplasmic fraction and to control for equal protein loading. B) Caspase-9 activity. * *P*<0.05 vs. the N group; # *P*<0.05 vs. the H group; n = 3.

To determine whether mitochondria-dependent apoptosis was involved, we detected mitochondrial and cytosolic proteins by western blotting. COXIV, a mitochondrial protein whose localization does not change on disruption of the mitochondrial outer membrane, was used to determine the purity of the cytoplasmic fraction and as a control for equal mitochondrial protein loading. GAPDH was used as the cytosolic loading control. As shown in Figure 3A, compared to the N group, leakage of the caspase activator cytochrome c from the mitochondria to the cytosol was attenuated in the H group. Puerarin enhanced the leakage of cytochrome c from the mitochondria in hypoxic HPASMCs when applied at 10, 50, and 100 µM after 24 h. However, puerarin did not affect cytochrome c distribution in normoxic HPASMCs.

Caspase-9 is an initiator caspase in the mitochondrial death pathway. We measured the enzymatic activity of caspase-9 by an *in vitro* colorimetric assay using cell lysates of HPASMCs treated with hypoxia or puerarin. As shown in Figure 3B, the activity of caspase-9 was suppressed in the H group compared to the N group. Puerarin increased caspase-9 activity in hypoxic HPASMCs in a dose-dependent manner, but had no effect on caspase-9 activity in normoxic cells.

### Puerarin decreased Bcl-2, but increased Bax expression, in hypoxic HPASMCs

**Figure 4:**
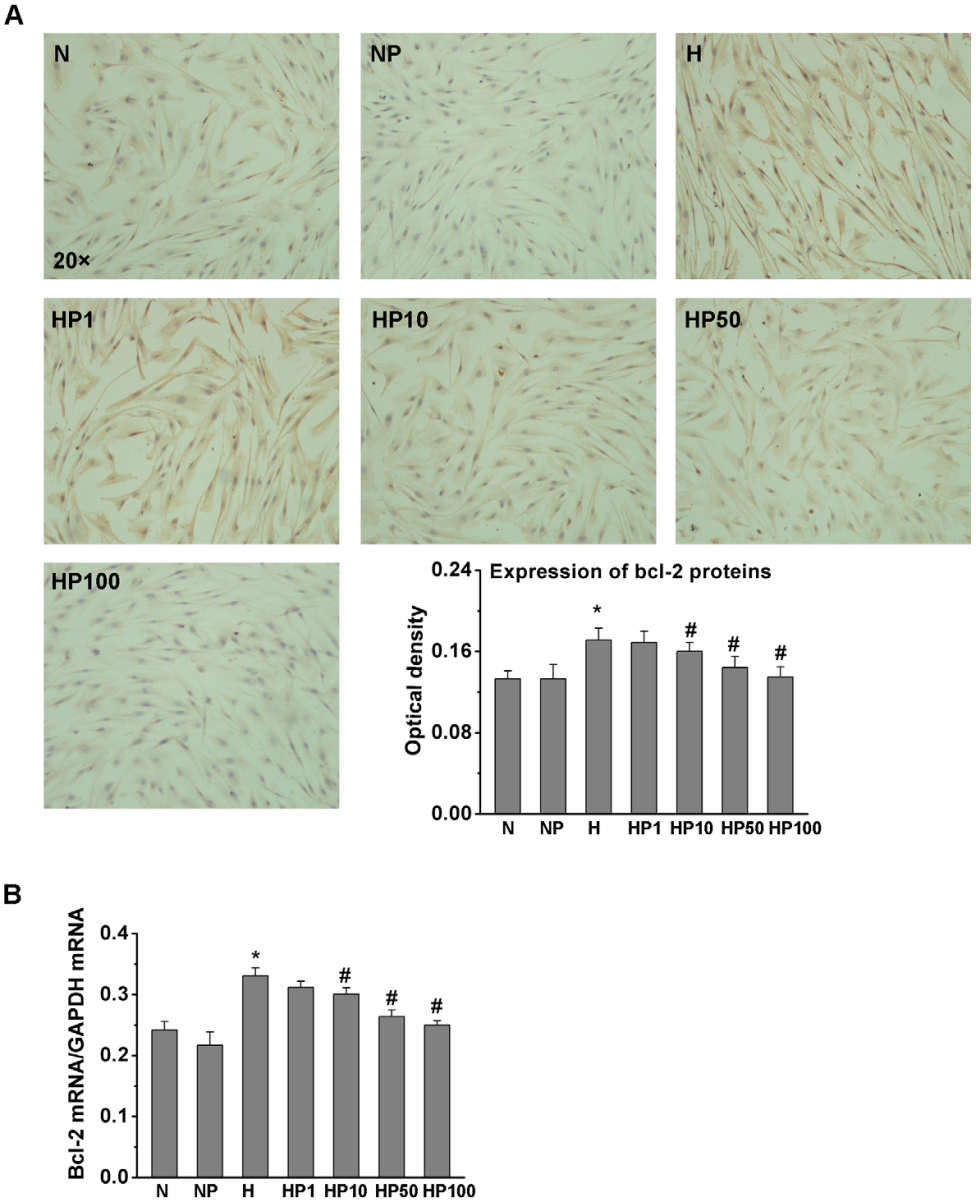
Puerarin decreased Bcl-2 expression in hypoxic HPASMCs. Treated HPASMCs were assayed for Bcl-2 protein (A) and mRNA (B). * *P*<0.05 vs. the N group; # *P*<0.05 vs. the H group; n = 3.

**Figure 5:**
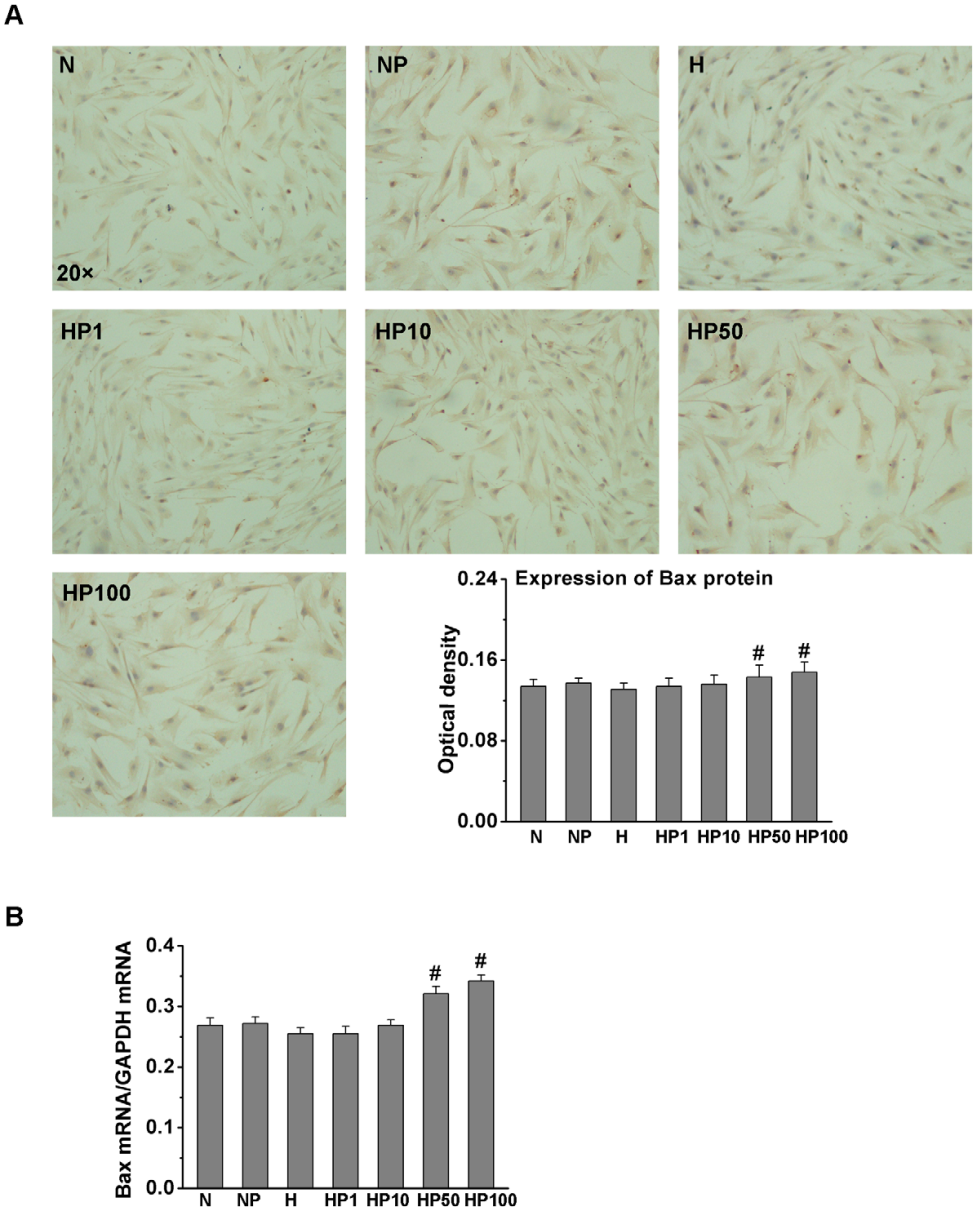
Puerarin increased Bax expression in hypoxic HPASMCs. Treated HPASMCs were assayed for Bax protein (A) and mRNA (B). * *P*<0.05 vs. the N group; # *P*<0.05 vs. the H group; n = 3.

Bcl-2 family member proteins mediate the mitochondrial apoptotic pathway. Therefore, we examined the effect of hypoxia and puerarin on Bcl-2 and Bax expression (Figure 4). The expression of Bcl-2 protein and mRNA significantly increased in the H group compared to the N group, while puerarin suppressed Bcl-2 protein and mRNA expression in hypoxic HPASMCs in a dose-dependent manner. Bax protein and mRNA expression (Figure 5) had no obvious changes in the H group compared to the N group. Puerarin (50 µM and 100 µM) induced Bax protein and mRNA expression in hypoxic HPASMCs. However, puerarin (50 µM) did not affect Bcl-2 and Bax expression significantly in normoxic HPASMCs.

## Discussion

Precise control of the balance of apoptosis and cell proliferation in PASMCs is critical in maintaining the normal structural and functional integrity of the pulmonary vasculature. The present study confirmed that hypoxia could not only induce HPASMC proliferation, but could also suppress apoptosis in these cells, which is in accordance with previous studies [18], [25]. We, for the first time, demonstrated that puerarin enhanced apoptosis (measured by the TUNEL assay) in hypoxic HPASMCs *in vitro* through a mechanism that involved the mitochondria-dependent pathway. Hypoxic HPASMCs had a significantly increased Δψ_m_, which contributed to the decrease of the mitochondrial permeability, further suppressing apoptosis and increasing HPASMCs viability. In addition, leakage of cytochrome c release to the cytosol was suppressed in hypoxic HPASMCs. Furthermore, caspase-9 activity was attenuated in hypoxic HPASMCs. Thus, we conclude that mitochondria-dependent apoptosis is suppressed in hypoxic HPASMCs. In contrast, puerarin depolarized HPASMC mitochondria and promoted the PT, the release of cytochrome c, and the activation of caspase-9. These results show that the mitochondria-dependent pathway is involved in puerarin-induced apoptosis.

The pro-apoptotic protein Bax can translocate to the outer membrane of mitochondria, change mitochondrial membrane permeability, and promote the loss of Δψm and cytochrome c release [26], [27]. On the other hand, Bcl-2, an anti-apoptotic member, can be sequestered in mitochondria, inhibiting the release of cytochrome c and preventing apoptosis. When interacting with activated pro-apoptotic proteins, the anti-apoptotic proteins lose their inhibiting activity, and apoptosis is initiated [28]. In our study, we showed that the ratio of Bax to Bcl-2 decreased in hypoxic HPASMCs and confirmed that apoptosis was suppressed in hypoxic HPASMCs. Xu and colleagues [29] found that 0.015–1.5 mM puerarin could significantly suppress Bcl-2 expression increased by thrombin in aortic smooth muscle cells. Additionally, Kang and colleagues [30] found that puerarin increased Bax levels and the Bax/Bcl-xl ratio in umbilical artery smooth muscle cells. In this study, at 50 or 100 µM, puerarin increased the ratio of Bax to Bcl-2 in hypoxic HPASMCs, which suggests that puerarin induces apoptosis in HPASMCs by modulating Bcl-2 family proteins upstream of the mitochondrial apoptotic pathway.

Our study also demonstrated that there were no significant effects on normal HPASMCs treated with puerarin at 50 µM for 24 h. Previous studies [31], [32] documented that upon administration of puerarin to chronic hypoxic rats, the mean pulmonary arterial pressure decreased markedly, but no significant change occurred in the mean carotid pressure. Additionally, puerarin protects various cell types from apoptosis [33]–[35], but induces apoptosis in some hyperproliferative cells, such as tumor cells and aortic smooth muscle cells stimulated by thrombin [22], [23], [29]. At present, we have no clear explanation for this pattern, but it is tempting to hypothesize that puerarin may target mitochondria only in proliferating HPASMCs, minimizing possible toxicity to healthy cells and tissues. However, further investigation is required.

In conclusion, our results show that puerarin can induce apoptosis, which is inhibited in hypoxic HPASMCs, and the mitochondria-dependent pathway is involved in puerarin-induced apoptosis. The present study provides a new theoretical basis for the use of puerarin in the management of hypoxic PH in the clinic.
